# Tubular iron deposition and iron handling proteins in human healthy kidney and chronic kidney disease

**DOI:** 10.1038/s41598-018-27107-8

**Published:** 2018-06-19

**Authors:** Sanne van Raaij, Rachel van Swelm, Karlijn Bouman, Maaike Cliteur, Marius C. van den Heuvel, Jeanne Pertijs, Dominic Patel, Paul Bass, Harry van Goor, Robert Unwin, Surjit Kaila Srai, Dorine Swinkels

**Affiliations:** 1 Department of Laboratory Medicine, Radboud Institute for Molecular Life Sciences, Radboud university medical center, Nijmegen, The Netherlands; 2 Department of Pathology and Medical Biology, University Medical Center Groningen and University of Groningen, Groningen, The Netherlands; 3 Pathologie Friesland, Leeuwarden, The Netherlands; 4 Department of Pharmacology and Toxicology, Radboud Institute for Molecular Life Sciences, Radboud university medical center, Nijmegen, The Netherlands; 5 Research Department of Pathology, UCL Cancer Institute, University College London, London, United Kingdom; 6 UCL Centre for Nephrology, Royal Free Hospital, London, United Kingdom; 7 Department of Cellular Pathology, Royal Free Hospital, London, United Kingdom; 8 Cardiovascular and Metabolic Diseases iMED ECD, AstraZeneca, Gothenburg, Sweden; 9 Department of Structural & Molecular Biology, Division of Biosciences, University College London, London, United Kingdom

## Abstract

Iron is suggested to play a detrimental role in the progression of chronic kidney disease (CKD). The kidney recycles iron back into the circulation. However, the localization of proteins relevant for physiological tubular iron handling and their potential role in CKD remain unclear. We examined associations between iron deposition, expression of iron handling proteins and tubular injury in kidney biopsies from CKD patients and healthy controls using immunohistochemistry. Iron was deposited in proximal (PT) and distal tubules (DT) in 33% of CKD biopsies, predominantly in pathologies with glomerular dysfunction, but absent in controls. In healthy kidney, PT contained proteins required for iron recycling including putative iron importers ZIP8, ZIP14, DMT1, iron storage proteins L- and H-ferritin and iron exporter ferroportin, while DT only contained ZIP8, ZIP14, and DMT1. In CKD, iron deposition associated with increased intensity of iron importers (ZIP14, ZIP8), storage proteins (L-, H-ferritin), and/or decreased ferroportin abundance. This demonstrates that tubular iron accumulation may result from increased iron uptake and/or inadequate iron export. Iron deposition associated with oxidative injury as indicated by heme oxygenase-1 abundance. In conclusion, iron deposition is relatively common in CKD, and may result from altered molecular iron handling and may contribute to renal injury.

## Introduction

Chronic kidney disease (CKD) affects 13% of the population worldwide^1^. Current treatment for CKD patients is mainly aimed at ameliorating renal symptoms, including proteinuria^2^, a major risk factor for disease progression^3^. However, in many patients, this does not prevent progression to end-stage renal disease^4^. The absence of targeted treatment modalities can, at least partly, be attributed to the lack of detailed molecular knowledge on the pathophysiological mechanisms of CKD.

Preclinical studies have suggested a detrimental role for (reactive) iron in the progression of CKD^5^. Increased exposure of renal tubular epithelial cells to iron leads to cellular damage^6–9^, since iron catalyzes highly reactive radical formation in the Fenton reaction^10^. In addition, in patients with various forms of CKD, increased urinary iron levels and renal iron deposition were found^8,11–19^, supporting an association between iron deposition and renal tubular injury. However, it has not been elucidated in which tubular segment iron is deposited.

Human-based studies examining renal iron handling are scarce^5^. It has been suggested that human renal tubular epithelial cells are able to handle iron in physiological conditions, but the localization of proteins involved in cellular iron handling is debated^20^. Transferrin-bound iron (TBI) in the systemic circulation is suggested to be filtered by the glomerulus into the tubular lumen^21,22^ and is subsequently completely reabsorbed by endocytic transport^20^. This can be facilitated by transferrin receptor 1 (TfR1) and the megalin-cubilin receptor complex in proximal tubular epithelial cells (PT), and the NGAL receptor (NGALR) in distal tubular epithelial cells (DT)^22–25^. Based on *in vitro* and *in vivo* studies, iron transport into the cytosol is reported to involve the putative divalent metal transporters ZIP8 (SLC39A8), ZIP14 (SLC39A14) or divalent metal transporter 1 (DMT1, SLC11A2)^26–28^. Subsequently, iron is oxidized by the ferroxidase H-ferritin and stored in L-ferritin, utilized by iron requiring processes, or exported into the blood stream by iron exporter ferroportin (SLC40A1)^20^.

Proteinuria as a result of glomerular damage in CKD is linked to tubulointerstitial injury^29^, which is associated with increased filtration of TBI^5,9,30^. Increased exposure of TBI can lead to tubular accumulation of reactive iron as a result of inadequate or disturbed iron handling. In diabetic nephropathy, the kidney can also be exposed to non-transferrin-bound iron (NTBI) derived from filtered TBI as a result of acidification of the filtrate as it passes along the nephron^5,31^, or directly filtered from the circulation^32^. NTBI uptake from the tubular lumen is thought to be mediated by ZIP8, ZIP14 and/or DMT1^20^.

For the first time in human kidney, we characterized associations between the presence and localization of iron deposition, proteins involved in cellular iron handling and tubular injury in kidney biopsies of patients with CKD and healthy controls.

## Results

### Iron deposition in CKD

**Figure 1:**
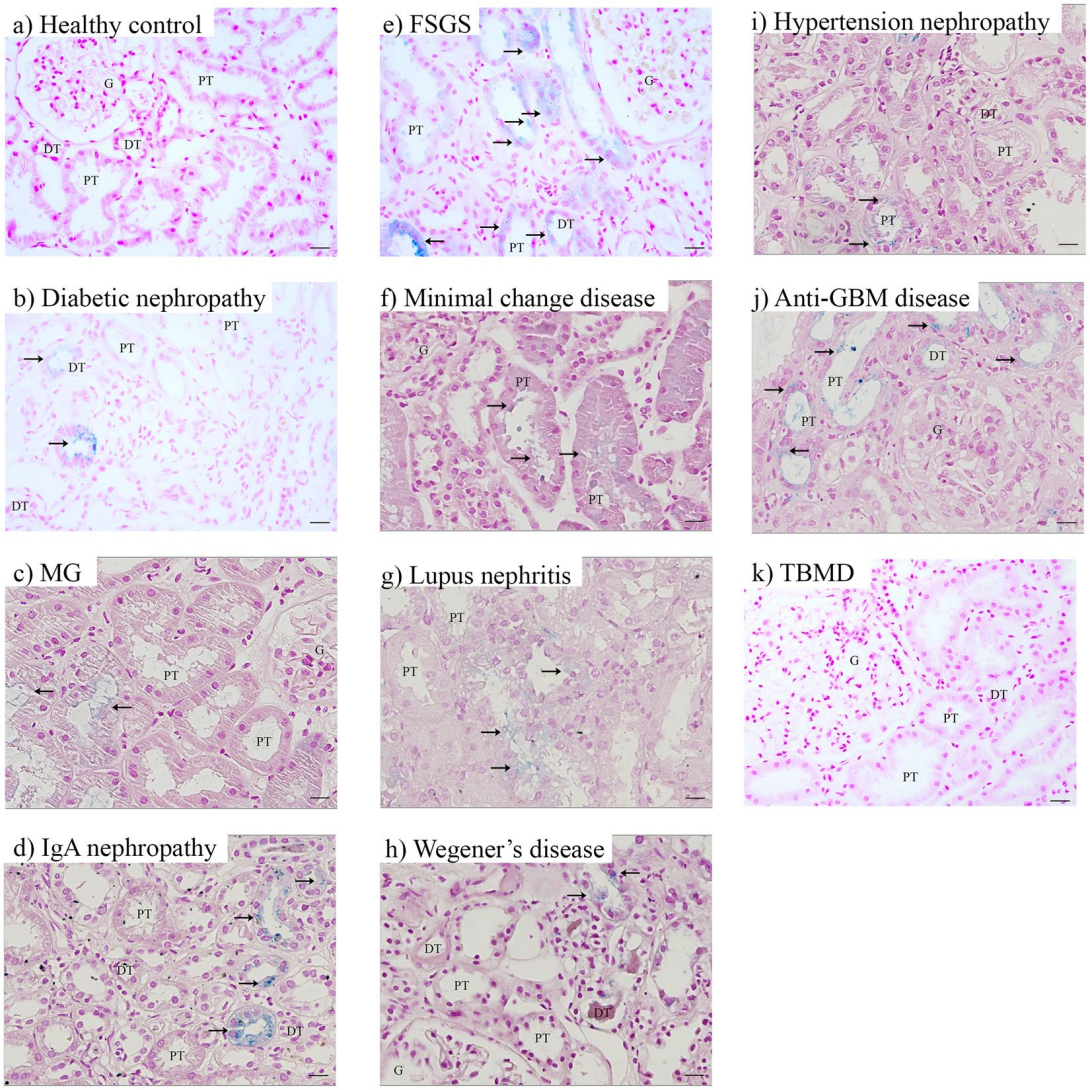
Iron deposition in chronic kidney disease. Representative images of Perls’ staining in healthy control (**a**), diabetic nephropathy (**b**), membranous glomerulopathy (MG; **c**), IgA nephropathy (**d**), focal segmental glomerulosclerosis (FSGS; **e**), minimal change disease (**f**), lupus nephritis (**g**), Wegener’s disease (**h**), hypertension nephropathy (**i**), anti-glomerular basement membrane (GBM) disease (**j**), and thin basement membrane disease (TBMD; **k**). Renal structures indicated as glomerulus (**G**), proximal tubule (PT), distal tubule (DT). Iron indicated with arrows. Scale bar 20 µM.

We found iron deposition in 33% of biopsies from various forms of CKD (n = 126), but not in controls (n = 8; Fig. 1, Table 1). Iron was deposited in a granular pattern in tubular epithelial cells and in the majority of CKD in both PT and DT. In minimal change disease, iron deposition was found in PT only. We detected iron deposition in kidney disorders characterized by nephrotic glomerulopathy (membranous glomerulopathy, focal segmental glomerular sclerosis (FSGS), minimal change disease), glomerulonephritis (Wegener’s disease, anti-glomerular basement membrane (GBM) disease), mesangial glomerular expansion (diabetic nephropathy), and potentially mixed nephrotic and nephritic glomerular injury (lupus nephritis (LN), IgA nephropathy (IgAN), hypertensive glomerulopathy) (Fig. 1b–k). These findings suggest that renal tubular iron deposition is a relatively common phenomenon in kidney disorders with glomerular dysfunction of different natures.

**Table 1:** Prevalence of tubular iron deposition in renal biopsies in chronic kidney disease.

Kidney disease	Abbreviation	Patients with tubular iron deposition/Total patients (n/n) (%)	Localization of iron deposition
Diabetic nephropathy	DN	6/27 (22)	PT + DT
Membranous glomerulopathy		7/21 (33)	PT + DT
IgA nephropathy	IgAN	7/19 (37)	PT + DT
Focal segmental glomerular sclerosis	FSGS	6/19 (32)	PT + DT
Minimal change disease		3/13 (23)	PT
Lupus nephritis	LN	3/11 (27)	PT + DT
Wegener’s disease		5/7 (71)	PT + DT
Hypertension nephropathy		3/4 (75)	PT + DT
Anti-glomerular basement membrane disease		1/1 (100)	PT + DT
Thin basement membrane disease		0/1 (0)	
Total		41/123 (33)	
Healthy control		0/8 (0)	

DT, distal tubule; PT, proximal tubule.

### Tubular iron handling proteins in healthy kidney

**Figure 2:**
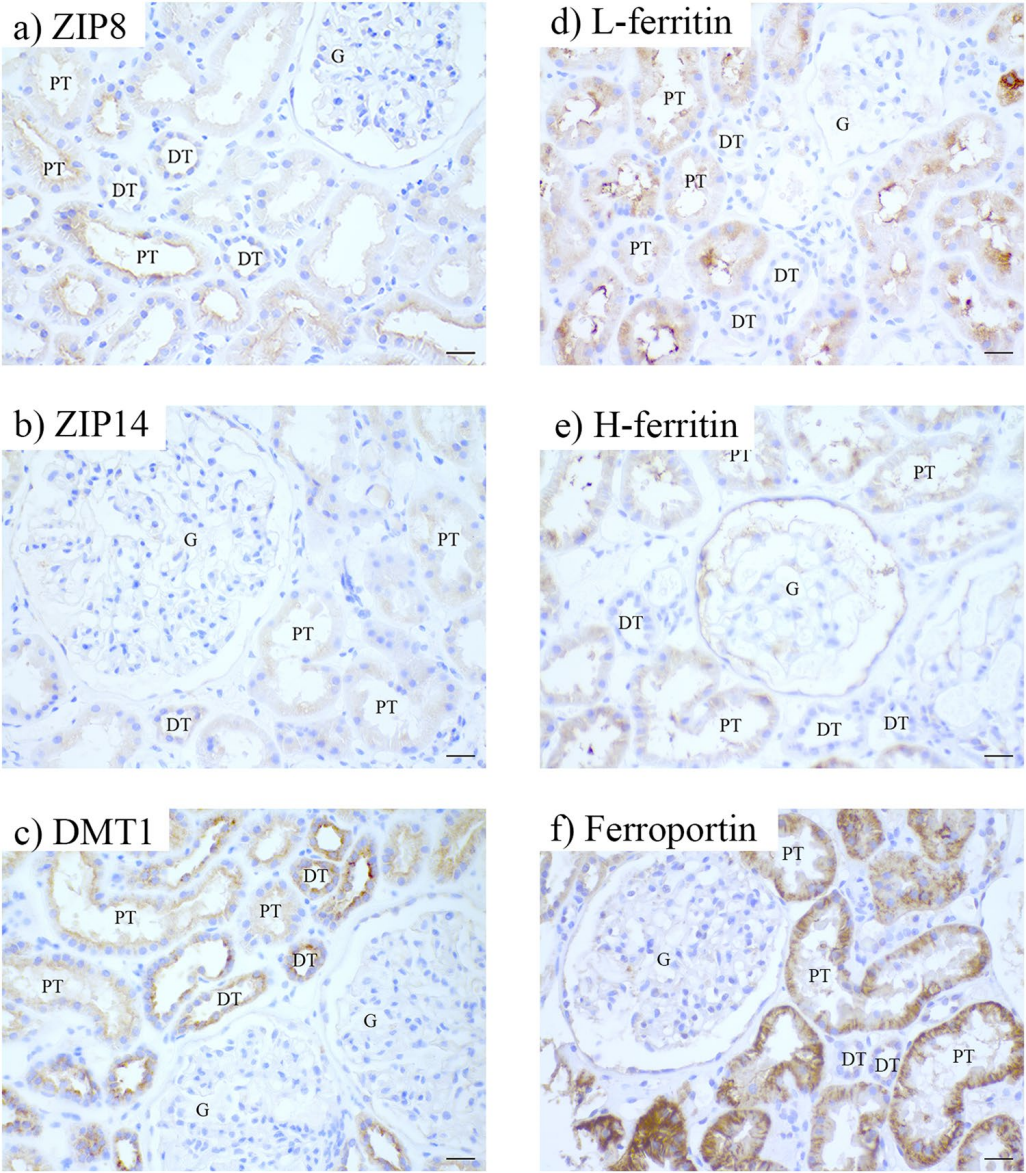
Immunohistochemistry of iron handling proteins in healthy kidney. Representatives images of ZIP8 (**a**), ZIP14 (**b**), divalent metal transporter 1 (DMT1; **c**), L-ferritin (**d**), H-ferritin (**e**), and ferroportin (**f**) staining in healthy kidney. Renal structures indicated as glomerulus (**g**), proximal tubule (PT), distal tubule (DT). Scale bar 20 µM.

In controls, ZIP8, ZIP14 and DMT1 were detected in both PT and DT (Fig. 2a–c, Table 2). ZIP8 was detected at the apical side of the tubules, whereas ZIP14 was localized intracellularly. DMT1 was observed both apically and intracellularly. Interestingly, L-ferritin, H-ferritin and ferroportin were observed only in PT (Fig. 2d–f, Table 2). Both ferritins showed intracellular orientation while ferroportin was expressed intracellularly and at the basolateral membrane. These findings indicate that PT express proteins for iron import, storage and export, while DT only express proteins involved in iron import in physiological conditions.

**Table 2:** Tubular localization of iron handling proteins in the healthy kidney.

Protein	Proximal tubule	Distal tubule	Tubular localization		
			Apical	Basolateral	Intracellular
ZIP8	+	+	+	−	−
ZIP14	+	+	−	−	+
DMT1	+	+	+	−	+
L-ferritin	+	−	−	−	+
H-ferritin	+	−	−	−	+
Ferroportin	+	−	−	+	+

DMT1, divalent metal transporter 1; +, present; −, not detected.

### Tubular iron handling proteins in CKD

**Figure 3:**
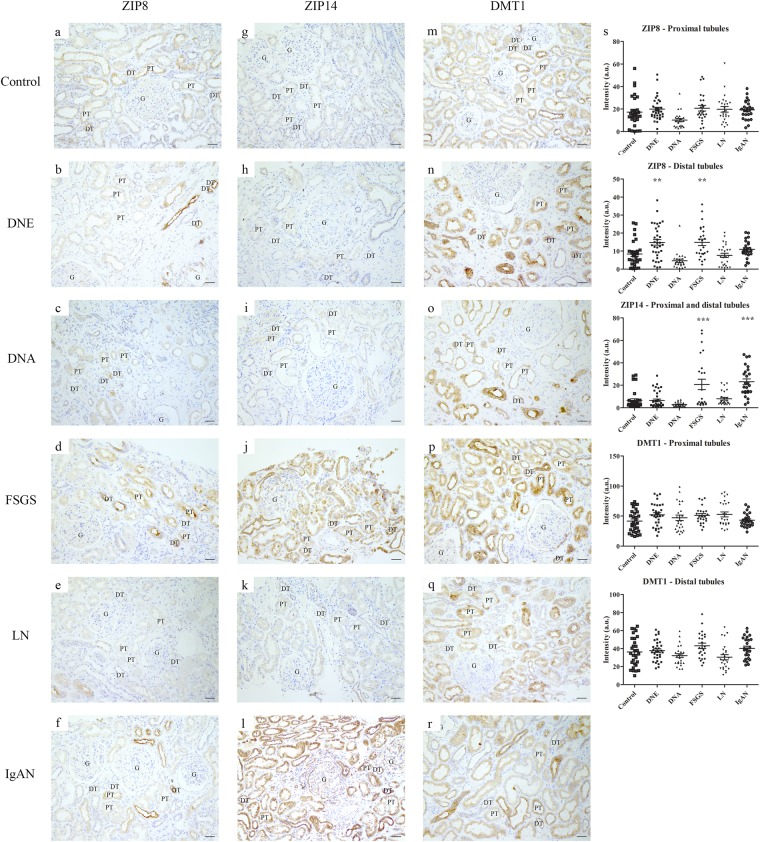
Immunohistochemistry of putative iron importers in chronic kidney disease. Representative images of ZIP8 (**a**–**f**), ZIP14 (**g**–**l**), and divalent metal transporter 1 (DMT1; **m**–**r**) staining in control (**a**,**g**,**m**), early diabetic nephropathy (DNE; **b**,**h**,**n**), advanced diabetic nephropathy (DNA; **c**,**i**,**o**), focal segmental glomerulosclerosis (FSGS; **d**,**j**,**p**), lupus nephritis (LN; **e**,**k**,**q**) and IgA nephropathy (IgAN; **f**,**l**,**r**). Intensity in proximal and distal tubules quantified (**s**). Dots represent all quantified images (5 images per biopsy). Renal structures indicated as glomerulus (G), proximal tubule (PT), distal tubule (DT). Scale bar 40 µM. **p < 0.01; ***p < 0.001.

**Figure 4:**
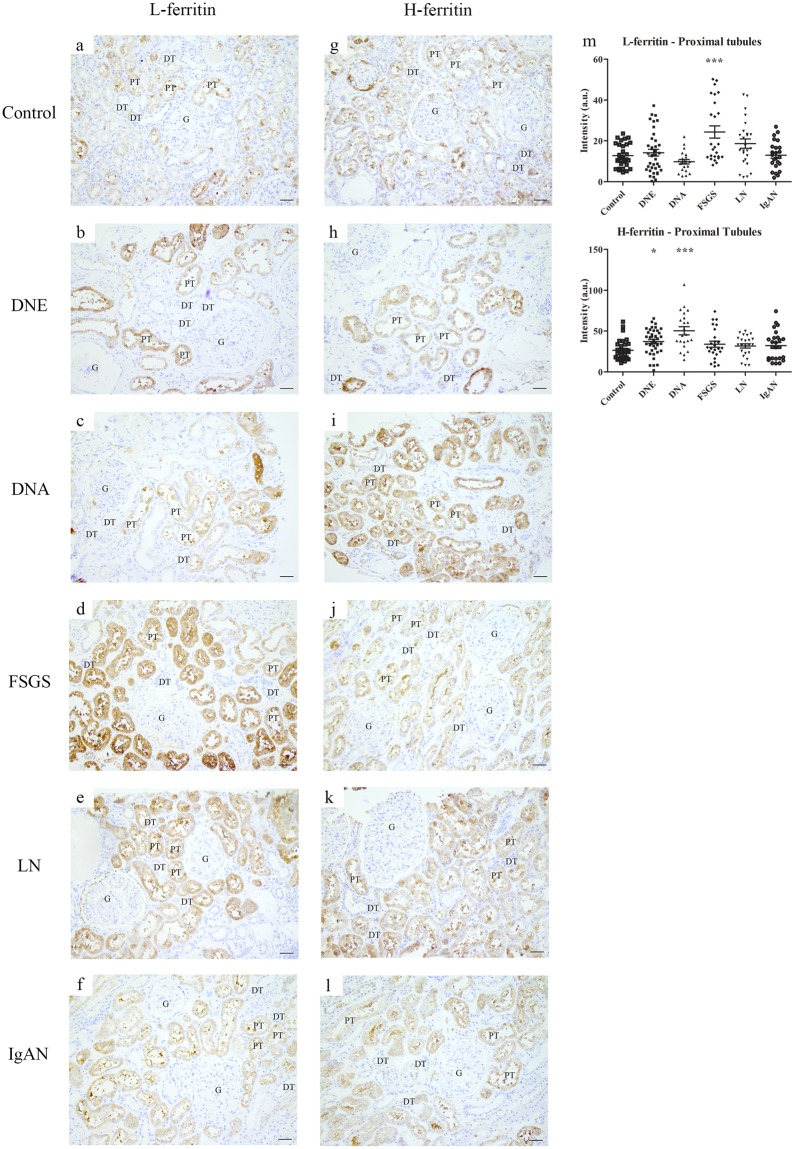
Immunohistochemistry of intracellular iron handling proteins in chronic kidney disease. Representative images of L-ferritin (**a**–**f**) and H-ferritin (**g**–**l**) staining in control (**a**,**g**), early diabetic nephropathy (DNE; **b**,**h**), advanced diabetic nephropathy (DNA; **c**,**i**), focal segmental glomerulosclerosis (FSGS; **d**,**j**), lupus nephritis (LN; **e**,**k**), and IgA nephropathy (IgAN; **f**,**l**). Intensity quantified (**m**) in proximal tubules. Dots represent all quantified images (5 images per biopsy). Renal structures indicated as glomerulus (G), proximal tubule (PT), distal tubule (DT). Scale bar 40 µM. *p < 0.05; ***p < 0.001.

**Figure 5:**
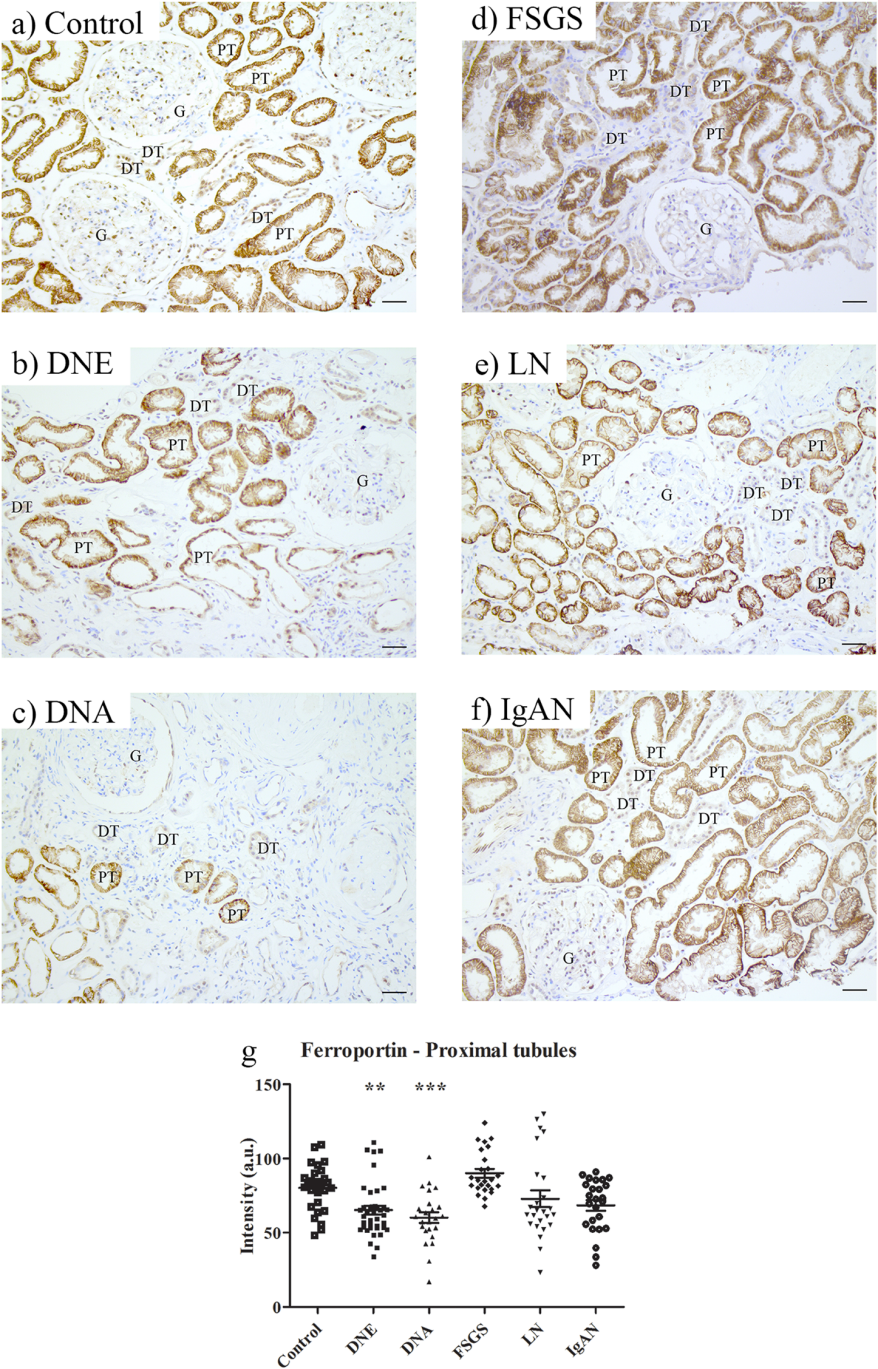
Immunohistochemistry of cellular iron export protein in chronic kidney disease. Representative images of ferroportin staining in control (**a**), early diabetic nephropathy (DNE; **b**), advanced diabetic nephropathy (DNA; **c**), focal segmental glomerulosclerosis (FSGS; **d**), lupus nephritis (LN; **e**), and IgA nephropathy (IgAN; **f**). Intensity quantified (**g**) in proximal tubules. Dots represent all quantified images (5 images per biopsy). Renal structures indicated as glomerulus (G), proximal tubule (PT), distal tubule (DT). Scale bar 40 µM. **p < 0.01; ***p < 0.001.

**Figure 6:**
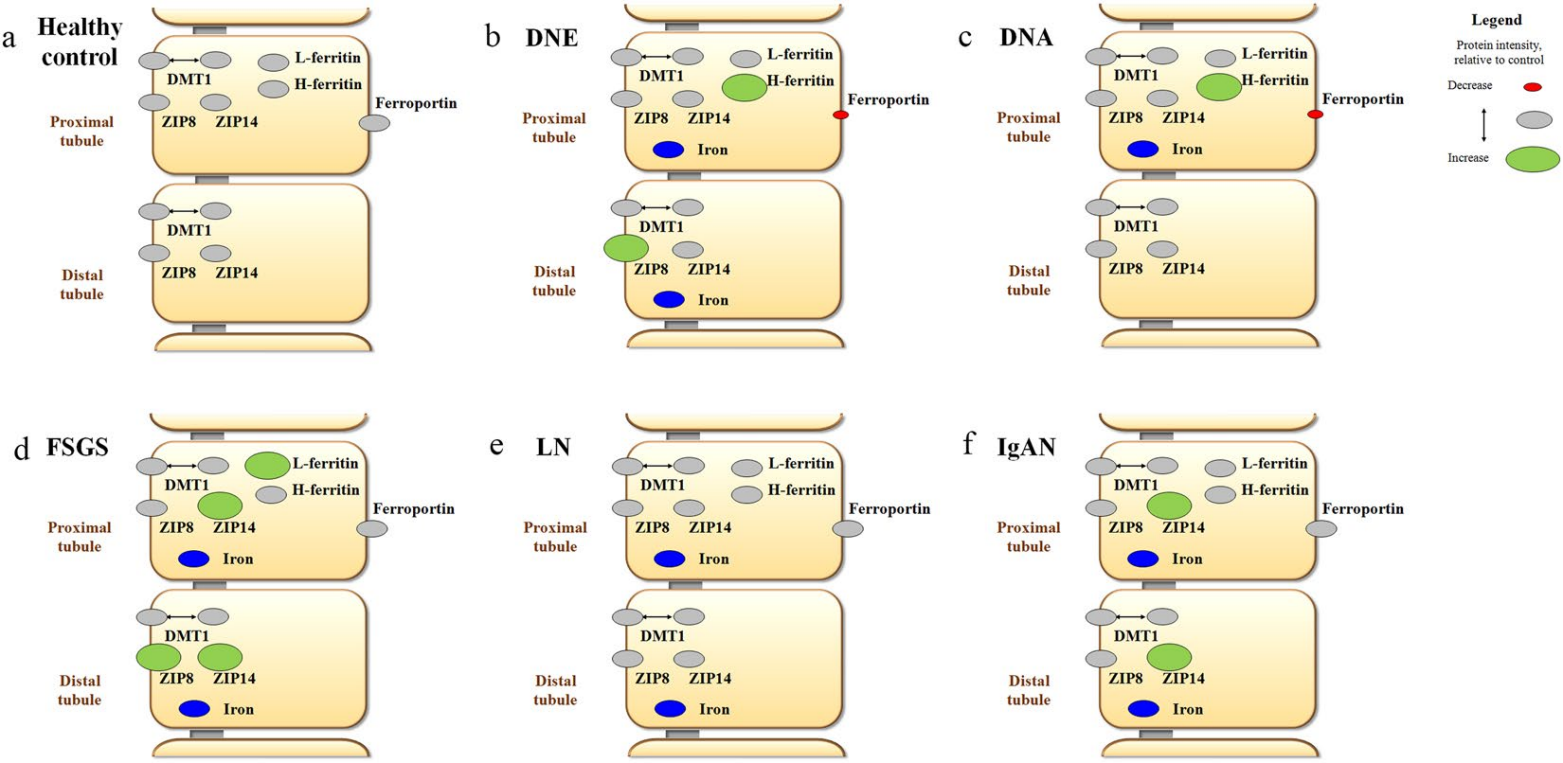
Overview of observations on iron deposition and intensity of iron handling proteins in chronic kidney disease. Overview of iron deposition (in blue) and iron handling protein intensity in healthy control (**a**), early diabetic nephropathy (DNE; **b**), advanced diabetic nephropathy (DNA; **c**), focal segmental glomerulosclerosis (FSGS; **d**), lupus nephritis (LN; **e**), and IgA nephropathy (IgAN; **f** ). Increased protein intensity compared to healthy controls (in grey) visualized in green, decreased protein intensity in red. *DMT1*, *divalent metal transporter 1*.

To characterize tubular iron handling during proteinuric CKD, a subset of kidney diseases was selected, including diabetic nephropathy, classified as early and advanced (DNE and DNA, respectively), FSGS, LN and IgAN (Table 3). Tubular localization of iron handling proteins in CKD was similar to controls (Figs 3–6). Staining intensity, however, differed between CKD and controls. ZIP14 showed comparable intensity in PT and DT, and, therefore, both segments were analyzed together. ZIP14 intensity was significantly increased in FSGS and IgAN compared to control (both p < 0.001; Fig. 3g–l,s). In FSGS, ZIP14 intensity was increased in two patients only, while all IgAN biopsies showed enhanced ZIP14 staining. ZIP8 and DMT1 showed differential staining between PT and DT, and were, therefore, analyzed separately. ZIP8 intensity was increased in DT in DNE and FSGS compared to control (both p < 0.01; Fig. 3a–f,s), while ZIP8 intensity in PT (Fig. 3a–f,s) was unchanged. DMT1 intensity in either PT or DT was not different from control (Fig. 3m–r,s). L-ferritin staining was increased in FSGS (p < 0.001; Fig. 4a–f,m), mainly caused by 2 biopsies, while H-ferritin was overall increased in DNE (p < 0.05) and DNA (p < 0.001; Fig. 4g–l,m). Moreover, ferroportin showed decreased intensity in DNE (p < 0.01) and DNA (p < 0.001) in PT (Fig. 5). Interestingly, for some CKD biopsies, the observed changes in iron handling protein intensity coincided with iron deposition. In FSGS and IgAN biopsies with PT iron deposition, ZIP14 intensity was increased, which was accompanied by increased L-ferritin intensity in FSGS. In DNE and DNA with PT iron deposition, H-ferritin abundance was increased along with decreased ferroportin intensity. In DT, ZIP8 and/or ZIP14 were increased concurrent with iron deposition in DNE, FSGS and IgAN, but neither were observed in DNA. In contrast, no changes in protein abundance of both tubules were seen in any of the LN biopsies despite iron deposition in 2 of the biopsies. These findings are summarized in Fig. 6.

**Table 3:** Characteristics of patients included for immunohistochemical staining of cellular iron handling proteins.

Kidney disease	Patients (n)	Age (years, mean ± SD)	Gender (M/F)
Control	6	44.8 ± 16.1	3/3
DNE	8	46.6 ± 13.3	4/4
DNA	5	58.4 ± 12.4	4/1
FSGS	5	49.0 ± 18.3	3/2
LN	5	35.2 ± 14.2	1/4
IgAN	5	26.6 ± 9.2	3/2

DNE, established diabetic nephropathy; DNA, advanced diabetic nephropathy; F, female; FSGS, focal segmental glomerulosclerosis; IgAN, IgA nephropathy; LN, lupus nephritis; M, male.

Biopsies selected based on tissue availability aiming at age- and gender-matched groups.

In conclusion, our findings show associations between tubular iron deposition and abundance of iron handling proteins in most types of CKD, which differ between specific pathologies. Overall, tubular iron deposition was related to increased iron import (ZIP8, ZIP14) in PT and DT, in some CKD biopsies accompanied by increased storage (ferritin) or decreased export (ferroportin) in PT.

### Tubular injury in CKD

**Figure 7:**
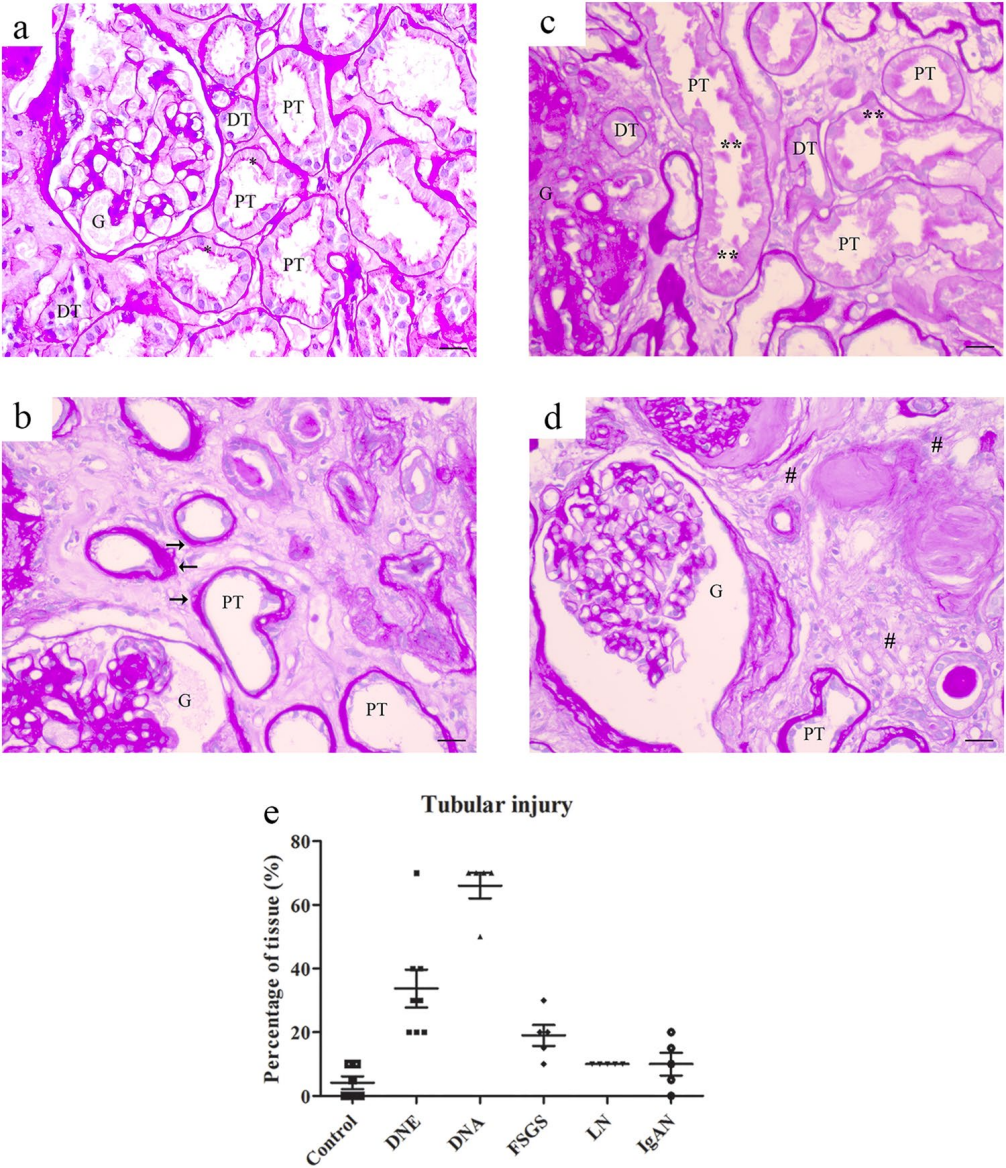
PAS staining for tubular injury in chronic kidney disease. Representative images of PAS staining in control (**a**) or chronic kidney disease showing atrophic tubules with loss of brush border and enlarged basement membrane (**b**), blebbed tubules (**c**) and interstitial fibrosis (**d**). Percentage of tissue with tubular injury scored (E) per patient. Renal structures indicated as glomerulus (G), proximal tubule (PT), and distal tubule (DT). Symbols indicate proximal tubular brush border with asterisk, blebbed tubules with double asterisk, basement membrane with arrow, and fibrosis with hashtag. Scale bar 20 µM.

**Figure 8:**
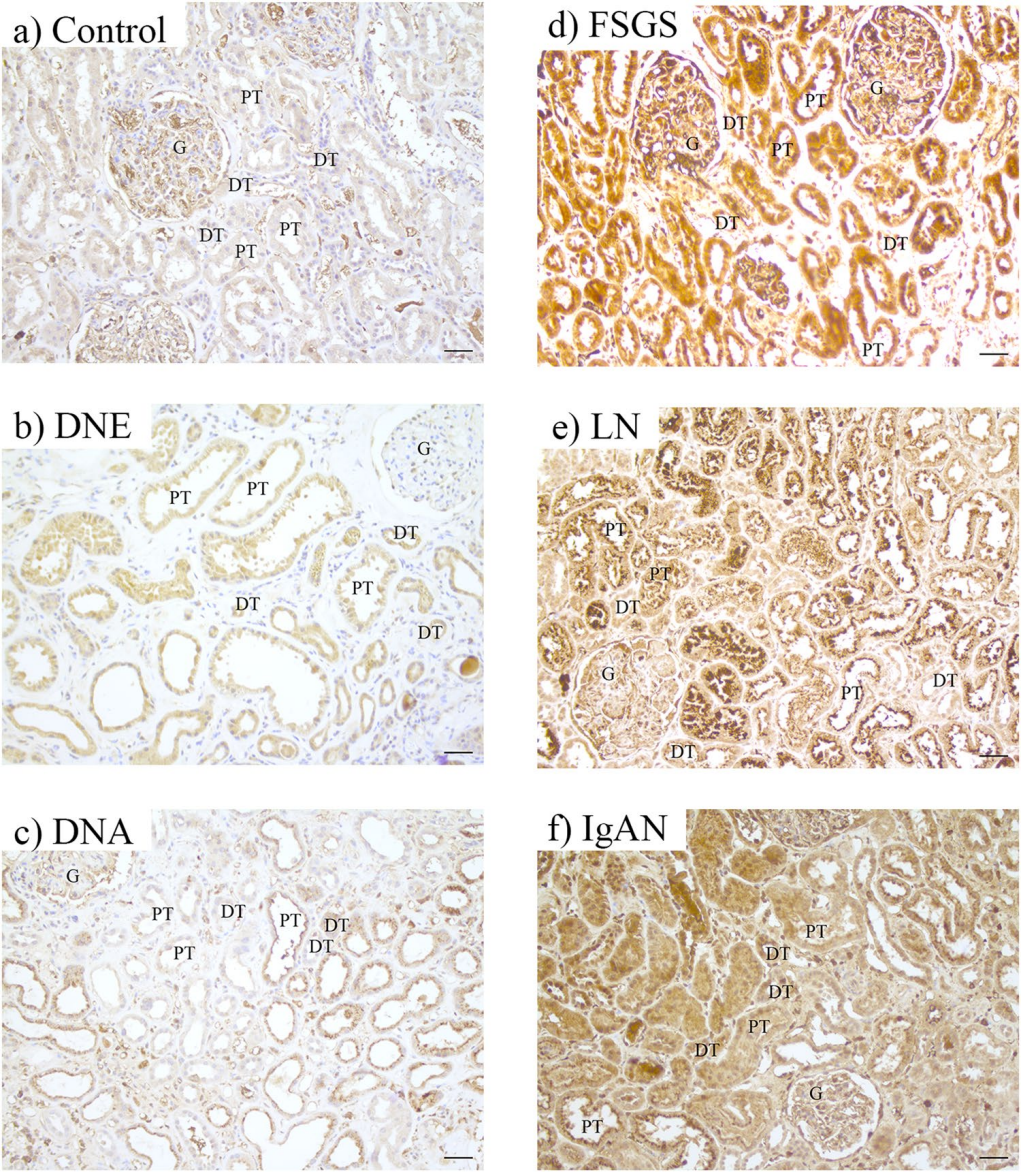
Immunohistochemistry of injury marker HO-1 in chronic kidney disease. Representative images of heme oxygenase-1 (HO-1) staining in control (**a**), early diabetic nephropathy (DNE; **b**), advanced diabetic nephropathy (DNA; **c**), focal segmental glomerulosclerosis (FSGS; **d**), lupus nephritis (LN; **e**), and IgA nephropathy (IgAN; **f**). Renal structures indicated as glomerulus (G), proximal tubule (PT), and distal tubule (DT). Scale bar 40 µM.

Tubular injury was assessed with PAS and heme oxygenase-1 (HO-1) staining. We used PAS staining to assess gross histology and scored renal injury based on tubular atrophy, blebbed tubular structures and irregular tubular cytoplasm (Fig. 7a–d). Renal damage was increased in DNE and DNA compared to control and moderately elevated in FSGS (Fig. 7e). HO-1, a marker for oxidative cellular stress^33^, showed comparable intensity in both PT and DT, which was increased in CKD (control < DNE = DNA < IgAN < FSGS = LN; Fig. 8). Increased HO-1 staining coincided with iron deposition in DNE, FSGS, LN and IgAN, but was also induced in DT of DNA without iron deposition.

In conclusion, we found oxidative cellular stress, but not histological damage, to associate with iron deposition.

## Discussion

Increased iron accumulation may be a detrimental factor in progression of CKD, but the mechanisms of iron handling in the human kidney are not fully elucidated. In this study we found iron deposition in one third of biopsies from various CKD disorders, predominantly in pathologies with glomerular dysfunction. We observed that in the healthy kidney, PT contain proteins involved in iron import, storage and export, while DT only showed proteins involved in iron import. Associations between iron deposition, intensity of iron handling proteins and tubular injury in CKD were seen, which differed between the pathologies. Nevertheless, in the majority of CKD, tubular iron deposition was accompanied by an increase in iron import proteins ZIP8 and/or ZIP14 in both PT and DT. This coincided with an increase in iron storage proteins or decrease in iron exporter in PT and increased oxidative cellular injury in both PT and DT.

Our data show that iron deposition is a relatively common phenomenon in CKD with glomerular dysfunction. In physiological conditions, TBI filtered by the glomerulus is believed to be almost completely reabsorbed by the tubular epithelium. In this process, PT reabsorb the bulk of filtered proteins and DT play only a minor role^20^. We hypothesize that in nephropathic glomerulopathy or pathologies with mesangial glomerular damage, PT iron reabsorption is increased by large amounts of TBI leaking from the injured glomerulus, resulting in local iron deposition. Moreover, when the reabsorption capacity of PT is overwhelmed, DT might also be exposed to increased iron levels, leading to DT iron deposition. In addition, we found iron deposition in pathologies with nephritic glomerular damage, which present with both hematuria and proteinuria^34^. Our results complement previous studies of renal iron deposition in CKD biopsies^11,12^, which was localized to PT lysosomes^18,19^. In our study, we demonstrated iron deposition also in DT in CKD. In addition, we show that iron deposition relates to oxidative cellular injury, as assessed by HO-1 staining. This strengthens the supposition that iron accumulation facilitates highly reactive radical formation that damage membranes, proteins and DNA, and, subsequently, causes tissue injury, which has been reported in renal tubular cells and animal models of CKD^6–9,35–37^. Interestingly, iron reduction via a low-iron diet or treatment with an iron chelator has been reported to reduce renal iron accumulation and tubulointerstitial injury in various CKD animal models^35–37^. Together with findings of increased urinary iron levels and renal iron deposition in patients with CKD^11–19^, this suggests that renal iron loading could contribute to disease progression in patients with CKD. Biopsies used in this study were taken for diagnostic purposes and are usually at a relatively early stage in disease progression, which is reflected by their tubular injury score. However, renal injury was already more established at the time of biopsy for diabetic nephropathy, especially for DNA. Nevertheless, our findings of iron deposition and associated oxidative cellular injury in FSGS, LN and IgAN biopsies suggest that iron may already be involved in the onset of renal disease and contribute to CKD progression.

We are the first to show ZIP14 and DMT1 in human kidney, whereas ZIP8 was previously described in human kidney^38^. Until now, DMT1 and ZIP14 were only demonstrated in murine PT, and DMT1 and ZIP8 in only mouse and rat DT^39–45^. Luminal TBI reabsorption is suggested to involve TfR1, but TfR1 localization, on the apical or basolateral membrane of tubular epithelial cells, is debated^31,41,46–48^. Since we did not obtain reliable TfR1 stainings, our study is not able to add to the understanding of TfR1 localization. Following entry in the cytosol, iron is known to be oxidized by H-ferritin and stored in L-ferritin. Our results of intracellular expression of both ferritins in PT agree with other studies^48,49^. Cytosolic iron is suggested to be transported back to the systemic circulation via the exporter ferroportin^20^. Although both apical and basolateral localization of ferroportin are shown in murine PT^31,41,46–48^, our human kidney results clearly demonstrate basolateral localization only. This discrepancy might be related to species difference^50^. In our studies, ferroportin was absent in DT, as is shown in rats^46^. The absence of ferritin and ferroportin in DT support that DT only play a minor role in physiological TBI reabsorption. Lack of effective iron storage and export could make these cells vulnerable to iron accumulation and related injury in case of high iron exposure.

For the majority of CKD disorders, we could distinguish an overall increased intensity of ZIP8 or ZIP14 that might have contributed to iron deposition. TfR1 and DMT1 are known to protect cells by limiting iron uptake via iron responsive element-iron responsive protein (IRE-IRP) regulation^51^, but this is not described for ZIP8 and ZIP14^52^. Interestingly, ZIP8 and ZIP14 increase with iron loading in hepatocytes^28,53^. Therefore, ZIP8 and ZIP14 are plausible candidates for unrestricted iron import and subsequent iron loading in kidney tubular epithelium from either endocytosis TBI or direct NTBI transport over the apical membrane^5,31,32^. Future assessment of the intracellular localization and function of ZIP8 and ZIP14 would be valuable to dissect potential iron transport routes in CKD.

We observed decreased ferroportin and concomitant increased H-ferritin intensity in diabetic nephropathy, which may explain PT iron deposition in these patients. Based on IRE-IRP regulation, however, we would expect ferroportin to increase with cellular iron loading^51^. At the systemic level, ferroportin is regulated by hepcidin, which causes ferroportin degradation^54^. Also renal ferroportin protein abundance is shown to decrease with high circulating hepcidin levels^55^. Interestingly, elevated hepcidin levels have been reported in patients with type 2 diabetes in the presence of chronic renal disease, obesity or inflammation^56,57^, which could explain the observed reduction in ferroportin intensity. Unfortunately, serum hepcidin levels were not available in our study. Moreover, renal biopsies in our study were obtained from patients with either type 1 or type 2 diabetes and the former patients do not have increased hepcidin levels^58^. Our findings of an association between decreased ferroportin and increased H-ferritin are corroborated by studies in human macrophages in which ferroportin silencing led to increased H-ferritin protein levels^59^, suggesting that H-ferritin upregulation could result from decreased ferroportin. Conversely, others showed a reciprocal relationship between H-ferritin and ferroportin by reporting reduced ferroportin mRNA and protein levels in conditional PT H-ferritin knockout mice^48^. Ferroportin is regulated by several multilayered signals, where hepcidin is reported to have a dominant effect^54^. This makes it difficult to determine whether renal iron deposition is due to ferroportin decreases or merely results from other mechanisms. Moreover, because of its multilayered regulation, the contribution of ferroportin to tubular iron loading may be different for various kidney diseases as observed in our study.

Besides ferroportin, also other cellular proteins involved in renal iron handling are regulated by processes beyond iron metabolism, including inflammation and oxidative stress^54,60,61^. These processes are also believed to contribute to the pathophysiology of CKD^62^. Therefore, changes in abundance of proteins we studied cannot solely be attributed to changes in iron handling. Vice versa, iron loading was observed in LN without changes in protein abundance. In these patients, iron accumulation could be mediated by other potential iron transporters that we were not able to include in this study, such as TfR1, the megalin-cubilin receptor complex or NGALR^22–25^. This underlines the complex mechanisms involved in the various CKD pathologies, which may (to various extents) all contribute to changes in renal iron handling proteins.

In summary, our findings in human renal biopsies form the basis for further elucidating renal iron handling in health and disease. Future studies should focus on unraveling the molecular mechanisms of renal iron loading in individual CKD disorders. This will determine whether and how reduction of renal iron accumulation in CKD is a feasible target to halt disease progression.

## Methods

### Design

Biopsies from patients with CKD and controls were used to examine the presence and localization of iron deposition. Subsequently, a selection of prevalent kidney diseases^63^ and controls was made to examine proteins involved in cellular iron handling and tubular injury.

### Patients

Renal biopsy material was collected from patients with CKD and potential kidney donors (healthy controls). Biopsies were classified based on clinical presentation of the patient and histological assessment by a trained renal pathologist. Biopsies were collected in University Medical Centre Groningen (the Netherlands (NL)) and Royal Free Hospital (RFH; London, United Kingdom (UK)), during the period of 1996–2014 and 2011–2016, respectively. Biopsies from both hospitals were used to assess iron deposition. For assessment of iron handling proteins and tubular injury, CKD biopsies and healthy kidneys from RFH only were selected, based on tissue availability aiming at age- and gender-matching. Biopsies with excessive tissue damage ( >70% of tissue) were excluded.

Procedures and use of anonymized material left over from diagnostic care were performed according to Dutch ethical guidelines. Accordingly, this study did not require approval from an ethical committee and material from patients from UMCG could be used without informed consent. All patients from RFH signed informed consent. All material was collected according to the Declaration of Helsinki.

### Histological stainings

Biopsy tissue was embedded in paraffin and cut into 5 µm sections. Renal histology was assessed by Periodic acid-Schiff (PAS) staining and iron deposition by Perls’ staining, according to routine staining protocols.

### Immunohistochemistry

Stainings were performed using the Bond Polymer Refine Detection system (Leica Biosystems, Newcastle-Upon-Tyne, UK) and provided materials on a Leica Bond Max and Autostainer XL apparatus for DMT1 (Novus Biologicals, Abingdon, UK, H00004891-M01, 1:2000), ZIP8 (Protein Tech, Manchester, UK, 20459-1-AP, 1:500), ZIP14 (Atlas Antibodies, Bromma, Sweden, HPA016508, 1:1000), L-ferritin (Abcam, Cambridge, UK, ab69090, 1:2000), H-ferritin (Abcam ab65080, 1:4000), ferroportin (Abcam ab85370, 1:300) and HO-1 (Abcam ab13243, 1:100). After antigen retrieval (Bond ER1 or ER2), sections were incubated with primary antibody for 15 min diluted in Bond Primary Antibody diluent or Antibody diluent with Background Reducing Components (Dako Agilent, Stockport, UK) and secondary antibody for 8 min. Signal visualization was performed with Polymer Refine for 8 min, DAB for 10 min and DAB enhancer (Leica Biosystems). Afterwards, nuclei were counterstained with haematoxylin and images were taken with a Leica DM 2000 microscope connected to a Leica Microsystem Ltd camera, NanoZoomer whole slide imager (Hamamatsu Photonics, Welwyn Garden City, UK) or a VisionTek digital microscope (Sakura Finetek, Alphen aan den Rijn, NL). Appropriate negative control stainings were included for all primary and secondary antibodies (Supplementary Figure 1 and 2, respectively).

### Image analysis

Stainings were evaluated by an expert in renal pathology. Staining intensity was assessed in 5 images per biopsy using the H DAB plugin of ImageJ after correction for background staining. Tubular injury was scored as percentage of the kidney section.

### Statistical analysis

Data were analyzed by one-way ANOVA with Dunnett’s post test using GraphPad Prism, to evaluate staining intensity and tubular injury in CKD compared to control. Differences were considered statistically significant when p < 0.05.

## Electronic supplementary material

Supplementary information 'Tubular iron deposition and iron handling proteins in human healthy kidney and chronic kidney disease'
