# Publisher Correction: Tubular iron deposition and iron handling proteins in human healthy kidney and chronic kidney disease

**DOI:** 10.1038/s41598-018-31457-8

**Published:** 2018-09-03

**Authors:** Sanne van Raaij, Rachel van Swelm, Karlijn Bouman, Maaike Cliteur, Marius C. van den Heuvel, Jeanne Pertijs, Dominic Patel, Paul Bass, Harry van Goor, Robert Unwin, Surjit Kaila Srai, Dorine Swinkels

**Affiliations:** 1 Department of Laboratory Medicine, Radboud Institute for Molecular Life Sciences, Radboud university medical center, Nijmegen, The Netherlands; 2 Department of Pathology and Medical Biology, University Medical Center Groningen and University of Groningen, Groningen, The Netherlands; 3 Pathologie Friesland, Leeuwarden, The Netherlands; 4 Department of Pharmacology and Toxicology, Radboud Institute for Molecular Life Sciences, Radboud university medical center, Nijmegen, The Netherlands; 5 Research Department of Pathology, UCL Cancer Institute, University College London, London, United Kingdom; 6 UCL Centre for Nephrology, Royal Free Hospital, London, United Kingdom; 7 Department of Cellular Pathology, Royal Free Hospital, London, United Kingdom; 8 Cardiovascular and Metabolic Diseases iMED ECD, AstraZeneca, Gothenburg, Sweden; 9 Department of Structural & Molecular Biology, Division of Biosciences, University College London, London, United Kingdom

Correction to: *Scientific Reports* 10.1038/s41598-018-27107-8, published online 19 June 2018

In the original version of this Article, the name of author Marius C. van den Heuvel was incorrectly given as Marius van den Heuvel.

In addition the authors Sanne van Raaij, Rachel van Swelm, Marius C. van den Heuvel and Harry van Goor were incorrectly indexed.

These errors have now been corrected in the PDF and HTML versions of the Article, and in the accompanying Supplementary Information file.

