# Supplementary material for: Tubular iron deposition and iron handling proteins in human healthy kidney and chronic kidney disease
Source: Sci Rep. 2018 Jun 19;8:9353. doi: 10.1038/s41598-018-27107-8 (PMC6008459; doi:10.1038/s41598-018-27107-8)
Supplement: Supplementary file 1 — Supplementary information 'Tubular iron deposition and iron handling proteins in human healthy kidney and chronic kidney disease' [file 41598_2018_27107_MOESM1_ESM.pdf]

Supplementary information

Tubular iron deposition and iron handling proteins in human healthy kidney and chronic kidney disease

Sanne van Raaij MSc[1], Rachel van Swelm PhD[1], Karlijn Bouman BSc[1], Maaike Cliteur BSc[1], Marius C. van den Heuvel MD, PhD[2,3], Jeanne Pertijs BSc[4], Dominic Patel MSc[5], Paul Bass MD[6,7], Harry van Goor MD, PhD[2], Robert Unwin MD, PhD[6,8], Surjit Kaila Srai PhD[9#*], Dorine Swinkels MD, PhD[1#*]

[1]Department of Laboratory Medicine, Radboud Institute for Molecular Life Sciences, Radboud university medical center, Nijmegen, the Netherlands; [2]Department of Pathology and Medical Biology, University Medical Center Groningen and University of Groningen, Groningen, the Netherlands; [3]Pathologie Friesland, Leeuwarden, the Netherlands; [4]Department of Pharmacology and Toxicology, Radboud Institute for Molecular Life Sciences, Radboud university medical center, Nijmegen, the Netherlands; [5]Research Department of Pathology, UCL Cancer Institute, University College London, London, United Kingdom; [6]UCL Centre for Nephrology, Royal Free Hospital, London, United Kingdom; [7]Department of Cellular Pathology, Royal Free Hospital, London, United Kingdom; [8]Cardiovascular and Metabolic Diseases iMED ECD, AstraZeneca, Gothenburg, Sweden; [9]Department of Structural & Molecular Biology, Division of Biosciences, University College London, London, United Kingdom; [#]contributed equally, [*]corresponding author.

Corresponding author: Dorine Swinkels, MD, PhD, Dorine.Swinkels@Radboudumc.nl, and Surjit Kaila Srai, PhD, k.srai@ucl.ac.uk.

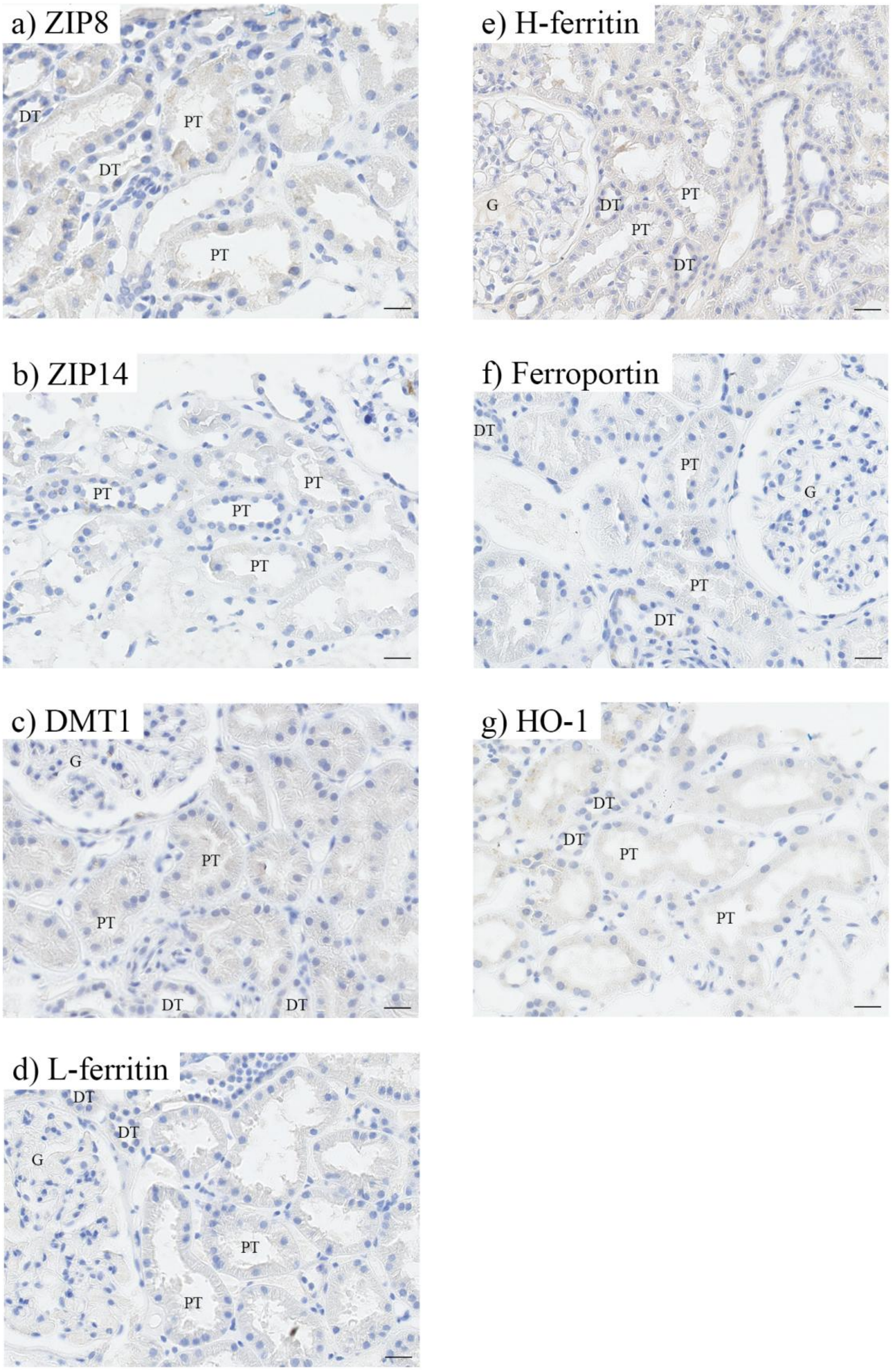

<u>Supplementary Figure 1: Primary antibody control stainings</u>

Representative images of primary antibody control (primary antibody replaced by IgG control) for staining procedure of ZIP8 (a), ZIP14 (b), divalent metal transporter 1 (DMT1; c), L-ferritin (d), H-ferritin (e), ferroportin (f) and heme oxygenase-1 (HO-1; g). Renal structures indicated as glomerulus (G), proximal tubule (PT), and distal tubule (DT). Scale bar 20 µM.

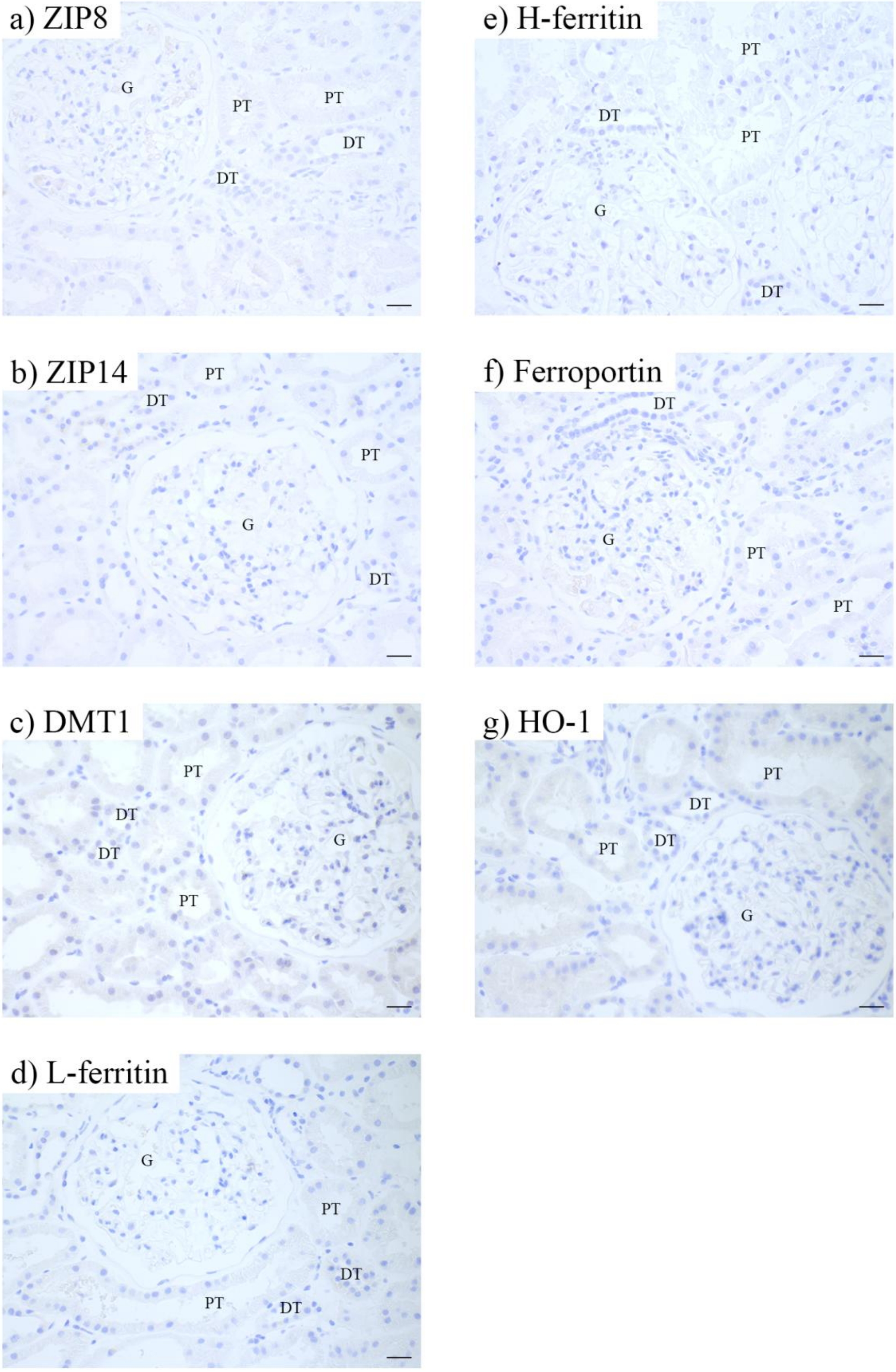

<u>Supplementary Figure 2: Secondary antibody control stainings</u>

Representative images of secondary antibody control (omission of primary antibody) for staining procedure of ZIP8 (a), ZIP14 (b), divalent metal transporter 1 (DMT1; c), L-ferritin (d), H-ferritin (e), ferroportin (f) and heme oxygenase-1 (HO-1; g). Renal structures indicated as glomerulus (G), proximal tubule (PT), and distal tubule (DT). Scale bar 20 µM.
